# Estimation and predictors of the Omega-3 Index in the UK Biobank

**DOI:** 10.1017/S0007114522003282

**Published:** 2023-07-28

**Authors:** Jan Philipp Schuchardt, Nathan Tintle, Jason Westra, William S. Harris

**Affiliations:** 1 The Fatty Acid Research Institute, Sioux Falls, SD, USA; 2 Institute of Food Science and Human Nutrition, Leibniz University Hannover, Hannover, Germany; 3 Department of Population Health Nursing Science, College of Nursing, University of Illinois – Chicago, Chicago, IL, USA; 4 Department of Internal Medicine, Sanford School of Medicine, University of South Dakota, Sioux Falls, SD, USA

**Keywords:** DHA, EPA, Erythrocyte, Fish intake, Predictor

## Abstract

Information on the Omega-3 Index (O3I) in the United Kingdom (UK) is scarce. The UK-Biobank (UKBB) contains data on total plasma *n*3-PUFA% and DHA% measured by NMR. The aim of our study was to create an equation to estimate the O3I (eO3I) from these data. We first performed an inter-laboratory experiment with 250 random blood samples in which the O3I was measured in erythrocytes by GC, and total *n*3 % and DHA% were measured in plasma by NMR. The best predictor of eO3I included both DHA% and a derived metric, the total *n*3 %–DHA%. Together these explained 65 % of the variability (*r* = 0·832, *P* < 0·0001). We then estimated the O3I in 117 108 UKBB subjects and correlated it with demographic and lifestyle variables in multivariable-adjusted models. The mean eO3I was 5·58 % (sd 2·35 %) in this UKBB cohort. Several predictors were significantly correlated with eO3I (all *P* < 0·0001). In general order of impact and with directionality (–, inverse and +, direct): oily-fish consumption (+), fish oil supplement use (+), female sex (+), older age (+), alcohol use (+), smoking (–), higher waist circumference and BMI (–), lower socioeconomic status and less education (–). Only 20·5 % of eO3I variability could be explained by predictors investigated, and oily fish consumption accounted for 7·0 % of that. With the availability of the eO3I in the UKBB cohort, we will be in a position to link risk for a variety of diseases with this commonly used and well-documented marker of *n*3-PUFA biostatus.

A large number of epidemiological, clinical and experimental studies have been conducted over the past few decades investigating the role of long-chain *n*3-PUFA for health. In particular, EPA (20:5) and DHA (22:6) are viewed to have beneficial effects for CVD^(1–3)^, cancer^(3,4)^, diabetes^(5,6)^, the metabolic syndrome^(7)^, dementia or Alzheimer’s disease^(8)^ and depression^(9)^. However, results from randomised trials exploring the potential protective nature of *n*3-PUFA on disease outcomes across this spectrum vary from beneficial to null^(10)^. Because of the inability of relatively short-term (years, not decades) intervention studies to reveal the adverse effects of subclinical nutritional deficiencies, a clearer picture of the relationships between *n*3-PUFA and risk for human disease may be achieved using prospective observational data based on *n*3-PUFA biomarkers rather than dietary intake data as the exposure.

The *n*3-PUFA biostatus can be assessed in a variety of lipid pools. First, *n*3-PUFA can be measured across numerous blood compartments including erythrocytes, whole plasma, whole blood, platelets, leukocytes and plasma lipid classes (i.e. phospholipids, cholesteryl esters, TAG and free FA). Erythrocytes are perhaps best suited to quantify long-term *n*3-PUFA blood levels since *n*3-PUFA in erythrocytes are constant over weeks and months compared with total plasma fatty acids or plasma phospholipid fatty acids where stability is only observed over days^(11)^. In addition, the *n*3-PUFA content of erythrocytes is similar to that of many organs including the heart, intestines and muscle, while FA in different compartments are less correlated with levels in these organs^(12)^.

The Omega-3 Index (O3I) – defined as the EPA + DHA content of erythrocytes as a percentage of total identified FA^(13)^ – has proven to be a suitable marker for measuring the *n*3-PUFA biostatus. In addition to the aforementioned reasons, it also has the lowest intra-individual variability compared with other markers such as plasma or plasma phospholipids. Originally proposed as a risk factor for fatal CHD, O3I risk categories were high (< 4 %), intermediate (4–6 %) and low (> 8 %)^(13)^.

Despite its importance, to our knowledge, only two countries have quantified *n*3-PUFA levels in formal national health surveys. The National Health and Nutrition Examination Survey in the USA reported plasma fatty acid levels as concentrations (µmol/l), whereas the Canadian Health Measures Survey used erythrocytes and reported national O3I levels^(14)^. The latter found that the mean O3I was comparatively low at 4·5 %. Data from other studies show that the average O3I is also in the low range in countries such as the USA, Italy or Germany and not as high as in Japan or South Korea, where mean O3I levels are in a desirable range. Comprehensive data on the O3I status are not available in the United Kingdom (UK); however, with data from the UK Biobank now available^(15,16)^, the *n*3-PUFA biostatus in that country may now theoretically be determined. However, the plasma FA data from the UK Biobank were expressed as concentrations or as a percentage of total plasma fatty acids, so how these metrics compare with the O3I is unclear.

The first aim of our study was to develop an equation to estimate the O3I (eO3I) from the NMR data of the UK Biobank so as to be able to compare UK O3I levels with those in other countries. The second aim was to examine the cross-sectional relations of the eO3I with important demographic (age, ethnic group, sex), anthropometric (BMI, waist circumference (WC)) and lifestyle (fish oil supplement use, (oily) fish consumption, alcohol use, smoking, etc.) factors to help define the determinants of the eO3I. To accomplish these aims, we first performed an inter-laboratory experiment to compare NMR-derived FA data with the O3I using GC-derived data. With a conversion equation thus generated, eO3I values were computed and then correlated in multivariable-adjusted models with demographic and lifestyle variables.

## Methods

### UK Biobank

UK Biobank is a prospective, population-based cohort of approximately 500 000 individuals recruited between 2007 and 2010. Baseline data were collected at twenty-two centres across England, Wales and Scotland^(17)^. Baseline data derived from questionnaires, biological samples and physical measurements were collected on all participating individuals, with longitudinal monitoring occurring via a mix of in-person and Electronic Medical Record data^(15,16)^. The participants completed a touchscreen questionnaire, which collected information on socio-demographic characteristics, diet and lifestyle factors. Anthropometric measurements were taken using standardised procedures. The touchscreen questionnaire and other resources are shown on the UK Biobank website (http://www.ukbiobank.ac.uk). UK Biobank has ethical approval (Ref. 11/NW/0382) from the North West Multi-centre Research Ethics Committee as a Research Tissue Bank. This approval means that researchers do not require separate ethical clearance and can operate under the Research Tissue Bank approval. All participants gave electronic signed informed consent. The UK Biobank study was conducted according to the guidelines laid down in the Declaration of Helsinki.

**Fig. 1.**
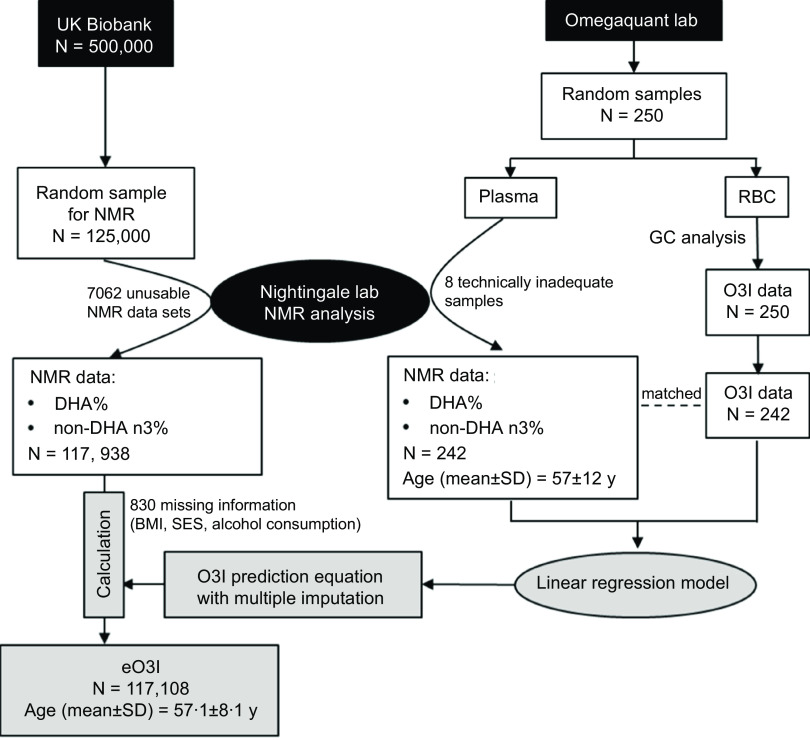
Flow chart for the inter-laboratory experiment to create an Omega-3 Index (O3I) prediction equation and calculation of the estimated Omega-3 Index (eO3I) from the UK Biobank data (for details see text). SES, socio-economic status.

A random sample of approximately 125 000 participants was selected for biomarker assessment using NMR (Nightingale Health Plc.)^(18)^ which included some plasma FA. Usable NMR data were available from 117 938 participants. After removing individuals with missing information on BMI, socio-economic status (SES) or alcohol use (*n* 830), the final sample size for this study was 117 108 (Fig. 1).

### Inter-laboratory experiment

An inter-laboratory experiment was undertaken to gather data for the creation of an O3I prediction equation (Fig. 1). Random blood samples (*n* 250) received at OmegaQuant Analytics) for routine testing of the O3I were used for this study. EDTA blood tubes were spun to isolate the erythrocyte fraction which was then analysed for the O3I by GC as previously described^(19)^. The plasma from these samples, which is normally discarded, was stored at –80°C. Once 250 had been collected (over about 2 weeks), the plasma aliquots were sent on dry ice for NMR analysis at the Nightingale lab. The data received from this analysis (*n* 242, 8 being technically inadequate) included multiple biometrics^(20)^ which were subsequently used as predictors of the O3I as described below.

### Statistical methods

#### Generating a prediction model for the estimated Omega-3 Index

As noted, the O3I is the sum of DHA and EPA in erythrocytes expressed as a percentage of total erythrocyte FA. The relevant NMR data for this study were plasma DHA and total *n*3-PUFA (each expressed as a percentage of total FA). No data on other *n*3-PUFA were available. Thus, we began by examining predictions of the O3I by total *n*3-PUFA%, DHA% and non-DHA *n*3-PUFA% (i.e. total *n*3-PUFA – DHA%). In order to maximise predictive ability and account for potential non-linearity and/or moderating effects, quadratic terms for each predictor plus an interaction term were evaluated for evidence of improved fit using a significance level of 0·05. The remaining 246 biometric measurements^(20)^ including 168 absolute levels and eighty-one ratio measures across cholesterol metabolism, amino acids, ketones, glycolysis metabolites and other fatty acids were then added to the model one at a time for evidence of improved fit, using a Bonferroni-adjusted significance level of 0·05/246 = 0·0002 as the criterion for addition to the predictive model. Three individuals with extreme DHA%/non-DHA% were temporarily excluded from the analyses to improve model fitting (two individuals with DHA% < 1 and one individual with DHA% > 4 and non-DHA% > 5 based on visual inspection of the boxplots (1·5 times IQR rule)) yielding a model-building sample size of 239. The final model was evaluated using *R* ^2^, residual standard error and the distribution of residuals. Values of the eO3I were then imputed by making stochastic draws from the predicted sampling distributions (final predictive model) as described by Rubin for data missing at random^(21)^. Sensitivity analyses were conducted considering extreme values of the observed DHA% and logarithmic transformations of the predictor and response variables to ensure robustness of the models to outliers and skewness^(22)^.

#### The estimated Omega-3 Index and participant characteristics

We used eO3I data generated as described above to investigate its relationship with fourteen sample characteristics. These variables were behaviour independent: demographic (sex, age, ethnic group, deprivation index, urbanicity, education), anthropometric (BMI, WC) and behaviour related: diet (frequency of oily and non-oily fish consumption), regular fish oil use, smoking, alcohol use and exercise (Table 1). BMI was classified according to the WHO into ‘normal weight’, ‘overweight’ and ‘obesity’. Since only 0·52 % of the cohort was underweight, this category was ignored. For WC categories, the cohort was divided into quartiles. No information on fish portion sizes or cooking methods, or on the EPA + DHA content of fish oil supplements (or dosage, potency and frequency of intake) was collected. Townsend deprivation index scores were derived from national census data about car ownership, household overcrowding, owner occupation and unemployment aggregated for residential postcodes^(23)^. Higher deprivation index scores indicate greater degrees of socio-economic deprivation. Our analysis used national quintiles of the deprivation index instead of continuous scores^(24)^. Four groups of education were formed: College (or University degree, other professional qualifications), Associates (A or AS level or equivalent), SE (O level, General Certificate of Secondary Education, Certificate of Secondary Education, National Vocational Qualifications, Higher National Diploma, Higher National Certificate or equivalent) and none. We analysed the bivariate relationships between the eO3I and each of the fourteen sample characteristics considering multiple imputations in the estimation of standard errors. A fully adjusted linear model predicted the eO3I by each of the fourteen sample characteristics simultaneously, again accounting for multiple imputation in the estimation of standard errors. Model *R* ^*2*^ was computed. In order to estimate the contribution of each predictor towards model *R* ^*2*^, each of the fourteen sample characteristics was then removed from the model (one at a time) and computing the difference in *R* ^*2*^ between the full and drop-one model. Parallel analyses were conducted for plasma percentage DHA (direct NMR measurement), without the need for multiple imputation. All analyses were conducted using standard statistical modelling functions with graphs from ggplot2, using R version 4.1.2. and used a 0·05 statistical significance threshold.

**Table 1.** Demographics of the UK Biobank sample (*n* 117 108) (Numbers and percentages; mean values and standard deviations)

Characteristic	%	*n*
Sex		
Female	54·21	63 480
Male	45·79	53 628
Age (years)		
Mean	57·1	
sd	8·1	
40–50 years	23·33	27 322
50–60 years	33·22	38 907
60–70 years	43·45	50 879
BMI (kg/m^2^)		
Mean	27·4	
sd	4·8	
18·5–24·9 (normal weight)	32·46	38 013
25·0–29·9 (overweight)	42·55	49 832
≥ 30 (obese)	24·47	28 656
Waist circumference (cm)		
< 80	22·81	26 717
80–90	26·28	30 777
90–99	25·14	29 439
> 99	25·74	30 145
Do not know/no answer	0·03	30
Ethnic group		
White	94·50	110 662
Black	0·57	662
Asian	1·93	2257
Other	3·01	3527
Deprivation index		
Mean	–1·3	
sd	3·1	
Least deprived	36·82	43 115
Next least deprived	21·19	24 819
Typical deprivation	14·59	17 090
Somewhat deprived	13·23	15 495
Most deprived	14·17	16 589
Urbanicity		
England/Wales – urban	85·6	100 250
England/Wales – rural	6·59	7722
Scotland – rural	0·66	773
Scotland – urban	6·18	7236
Education		
College	37·57	43 992
Associates	10·99	12 869
SE	33·28	38 969
None	17·1	20 023
Do not know/no answer	1·07	1255
Oily fish consumption		
Never	10·86	12 715
Less than 1×/week	32·75	38 356
1×/week	37·83	44 304
At least 2×/week	17·97	21 042
Non-oily fish consumption		
Never	4·71	5511
Less than 1×/week	28·75	33 668
1×/week	49·49	57 958
At least 2×/week	16·52	19 347
Regular fish oil consumption		
No	68·43	80 142
Yes	31·31	36 669
Alcohol use		
Rarely/never	19·36	22 667
1–3×/month	11·28	13 208
1–2×/week	25·82	30 233
3–4×/week	23·27	27 254
Daily	20·28	23 746
Smoker		
Never	54·37	63 671
Previous	34·7	40 632
Current	10·55	12 350
Exercise (min/week)		
< 150	22·12	25 901
150–320	22·81	26 710
320–660	22·92	26 846
> 660	23·17	27 130
Do not know/no answer	8·98	10 521

## Results

### Generating a prediction equation based on the inter-laboratory experiment

**Fig. 2.**
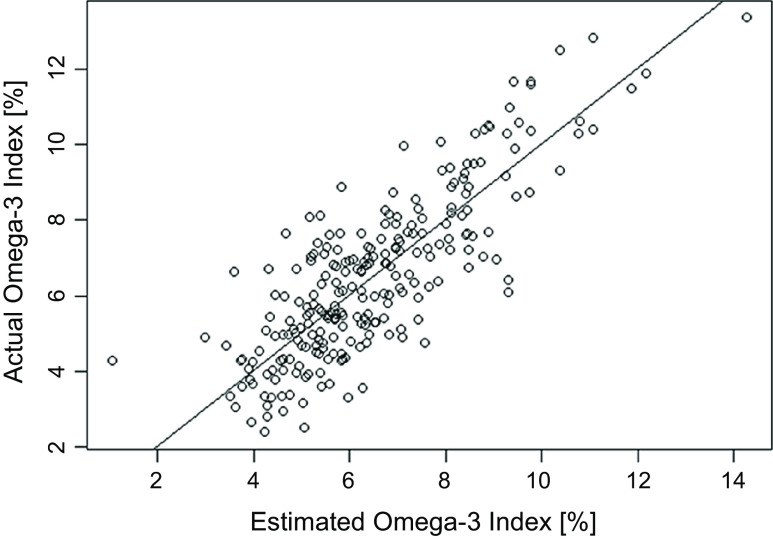
Predicted Omega-3 Index (eO3I) *v*. actual Omega-3 Index (O3I). The line of identity (y = x) is plotted. All values are percentage of total erythrocyte FA. Final *r* = 0·823.

In models predicting the O3I by a single variable, percentage DHA was a better predictor (*r* ^*2*^ = 0·65) than total *n*3 (*r* ^*2*^ = 0·60). We then added non-DHA% to the model with DHA%, yielding a model with *r* ^*2*^ = 0·67. Residuals showed a reasonably normal distribution (online Supplementary Fig. 1). Quadratic terms and an interaction between DHA% and non-DHA% were added to the model, but none showed evidence of improvement (*P* = 0·41 (quadratic DHA%); *P* = 0·80 (quadratic non-DHA%); *P* = 0·20 (interaction DHA and non-DHA%)). Log-transforming the O3I and/or DHA% and non-DHA% prior to fitting the regression equation did not improve the *r* ^*2*^ values (61–65 %) with little visible improvement in the normality of outliers (details not shown). Thus, the untransformed, additive model with two terms was determined to be the final prediction model (Table 2) which includes *β*-coefficients for term: eO3I = 2·629 × NMR DHA% + 0·4673 × NMR non-DHA% − 0·1014. The correlation of the eO3I *v*. observed O3I values was 0·823 on the complete dataset of *n* 242, including three extreme values which were not included in the initial model-building step (Fig. 2).

**Table 2.** Regression model predicting the Omega-3 Index (*n* 239) (Beta-coefficients and standard errors)

Predictor	*β*	se	*P*
Intercept	–0·1014	0·315	0·75
NMR DHA%	2·6290	0·194	< 2 × 10^−16^
NMR non-DHA%	0·4673	0·126	0·00027

Model summary statistics: residual SE (1·217); *r* ^*2*^ = 0·673; *r* = 0·823.

### Demographics of the UK Biobank sample

The demographics of the analysed UK Biobank sample are in Table 1. The cohort had a mean age of 57 years with slightly more women than men. The individuals were overwhelmingly White and lived in urban areas. The mean deprivation index was −1·3, meaning that, on average, this sample was somewhat less deprived than typical in the UK (which would be an index of 0). The plurality of individuals was overweight, followed by normal weight and then obese.

Most individuals in the cohort stated that they regularly ate fish. Non-oily fish is slightly preferred compared with oily fish. Almost half of the individuals stated that they ate non-oily fish 1×/week. However, the proportion of individuals who consumed oily fish 1×/week was also quite high at 38 %. Regular fish oil supplement use was reported by 31 %. Most of the study participants reported some regular alcohol use. More than half of the individuals were non-smokers, some were ex-smokers and a few continued to smoke.

### Predicted Omega-3 Index in the UK Biobank sample

An inter-laboratory experiment was undertaken in which 250 samples were analysed using different methods (plasma DHA% and non-DHA% by NMR; erythrocyte O3I by GC) at different laboratories (Nightingale and OmegaQuant Analytics). In this experiment, the mean of plasma DHA% from the NMR analysis was 2·1 % (sd 0·6 %) while the plasma non-DHA% was 2·1 % (sd 0·9 %). The mean GC-based O3I was 6·5 % (sd 2·2 %). All three measurements showed modest right skewness.

The mean eO3I was 5·58 % for all 117 938 individuals in this UK Biobank cohort. We used multiple (i.e. 10) imputations for each person using the prediction equation in Table 2 in order to compute the SD. The sd ignoring the multiple imputation step was 1·89 %, whereas with imputations it was 2·25 %, reflecting the variability from different analytical methods (NMR *v*. GC), different sample types (plasma *v*. erythrocyte) and different *n*3-PUFA (DHA *v*. EPA + DHA).

### Correlates of the estimated Omega-3 Index in the UK Biobank sample (Table 3)

**Table 3.** Associations of demographic characteristics and the estimated Omega-3 Index (eO3I) in unadjusted and adjusted (for all variables in Table 1) analyses (*n* 117 108) (Mean values and standard deviations; 95 % confidence intervals)

Characteristics	eO3I Mean	sd	eO3I unadj diff or corr	95 % CI	Unadj *P*	eO3I adj diff or corr	95 % CI	Adj *P*
Sex								
Female	5·9	2·2	0			0		
Male	5·2	2·2	–0·62	–0·65,–0·59	< 0·0001	–0·38	–0·42,–0·34	< 0·0001
Age (years)								
40–50 (reference)	5·2	2·1	0			0		
50–60	5·5	2·2	0·29	0·25,0·33	< 0·0001	0·15	0·11,0·19	< 0·0001
60–70	5·8	2·3	0·64	0·6,0·68	< 0·0001	0·35	0·31,0·39	< 0·0001
BMI (kg/m^2^)								
18·5–24·9 (normal weight; reference)	6	2·3	0			0		
25·0–29·9 (overweight)	5·6	2·2	–0·4	–0·43,–0·36	< 0·0001	–0·13	–0·18,–0·09	< 0·0001
≥ 30 (obese)	5·1	2·1	–0·88	–0·92,–0·84	< 0·0001	–0·38	–0·44,–0·32	< 0·0001
Waist circumference (cm)								
< 80 (reference)	6·2	2·3	0			0		
80–90	5·8	2·2	–0·38	–0·42,–0·33	< 0·0001	–0·22	–0·27,–0·17	< 0·0001
90–99	5·4	2·2	–0·72	–0·77,–0·67	< 0·0001	–0·37	–0·43,–0·31	< 0·0001
> 99	5·0	2·1	–1·13	–1·18,–1·08	< 0·0001	–0·55	–0·63,–0·47	< 0·0001
Do not know/no answer	4·9	1·7	–1·23	–2·24,–0·21	0·018	–0·8	–1·78,0·17	0·1
Ethnic group								
White (reference)	5·6	2·2	0			0		
Black	5·7	2·3	0·09	–0·12,0·31	0·41	0·17	–0·04,0·38	0·12
Asian	5	2·3	–0·58	–0·7,–0·46	< 0·0001	0	–0·12,0·12	0·97
Other	6·5	2·6	0·94	0·85,1·04	< 0·0001	1·03	0·93,1·12	< 0·0001
Deprivation index								
Least deprived (reference)	5·7	2·2	0			0		
Next least deprived	5·6	2·2	–0·09	–0·13,–0·04	0·0001	–0·01	–0·06,0·03	0·49
Typical Deprivation	5·5	2·2	–0·2	–0·25,–0·15	< 0·0001	–0·07	–0·12,–0·02	0·003
Somewhat deprived	5·4	2·2	–0·33	–0·38,–0·28	< 0·0001	–0·12	–0·17,–0·07	< 0·0001
Most deprived	5·3	2·3	–0·42	–0·48,–0·37	< 0·0001	–0·12	–0·18,–0·07	< 0·0001
Urbanicity								
England/Wales – urban (reference)	5·6	2·2	0			0		
England/Wales – rural	5·8	2·2	0·22	0·16,0·28	< 0·0001	0·02	–0·04,0·08	0·5
Scotland – rural	5·6	2·3	0·01	–0·19,0·2	0·96	0·03	–0·15,0·21	0·74
Scotland – urban	5·5	2·2	–0·1	–0·16,–0·03	0·0027	–0·06	–0·12,0	0·049
Education								
College (reference)	5·8	2·3	0			0		
Associates	5·7	2·2	–0·14	–0·2,–0·09	< 0·0001	–0·08	–0·13,–0·03	0·00094
SE	5·4	2·2	–0·37	–0·41,–0·34	< 0·0001	–0·18	–0·22,–0·15	< 0·0001
None	5·4	2·2	–0·42	–0·47,–0·38	< 0·0001	–0·23	–0·27,–0·18	< 0·0001
Do not know/no answer	5·4	2·2	–0·39	–0·54,–0·23	< 0·0001	–0·26	–0·4,–0·11	0·00056
Oily fish consumption								
Never (reference)	4·3	1·7	0			0		
Less than 1×/week	5	1·9	0·77	0·71,0·82	< 0·0001	0·47	0·41,0·53	< 0·0001
1×/week	5·8	2·1	1·56	1·51,1·62	< 0·0001	1·11	1·05,1·17	< 0·0001
At least 2×/week	6·9	2·6	2·64	2·57,2·7	< 0·0001	2·12	2·05,2·18	< 0·0001
Non-oily fish consumption								
Never (reference)	4·3	2	0			0		
Less than 1×/week	5·3	2·2	1	0·92,1·07	< 0·0001	0·34	0·26,0·42	< 0·0001
1×/week	5·7	2·2	1·38	1·31,1·46	< 0·0001	0·35	0·26,0·43	< 0·0001
At least 2×/week	6·1	2·4	1·79	1·71,1·87	< 0·0001	0·49	0·4,0·58	< 0·0001
Regular fish oil consumption								
No (reference)	5·3	2·1	0			0		
Yes	6·2	2·3	0·97	0·93,1	< 0·0001	0·68	0·65,0·71	< 0·0001
Alcohol use								
Rarely/never (reference)	5·4	2·3	0			0		
1–3×/month	5·4	2·2	–0·02	–0·08,0·04	0·61	0·01	–0·05,0·07	0·69
1–2×/week	5·5	2·2	0·14	0·09,0·19	< 0·0001	0·11	0·06,0·16	< 0·0001
3–4×/week	5·8	2·2	0·38	0·33,0·43	< 0·0001	0·26	0·21,0·31	< 0·0001
Daily	5·8	2·2	0·4	0·35,0·45	< 0·0001	0·3	0·25,0·35	< 0·0001
Smoker								
Never (reference)	5·7	2·3	0			0		
Previous	5·6	2·3	–0·04	–0·08,–0·01	0·025	–0·03	–0·07,–0	0·07
Current	4·8	2	–0·86	–0·92,–0·8	< 0·0001	–0·58	–0·64,–0·53	< 0·0001
Exercise (min/week)								
< 150 (reference)	5·5	2·2	0			0		
150–320	5·7	2·2	0·23	0·18,0·27	< 0·0001	0·01	–0·03,0·05	0·68
320–660	5·7	2·3	0·26	0·21,0·3	< 0·0001	–0·04	–0·08,0·01	0·092
> 660	5·6	2·3	0·12	0·07,0·16	< 0·0001	–0·16	–0·21,–0·12	< 0·0001
Do not know/no answer	5·3	2·2	–0·17	–0·17,–0·04	0·00086	–0·1	–0·16,–0·04	0·00074

**Fig. 3.**
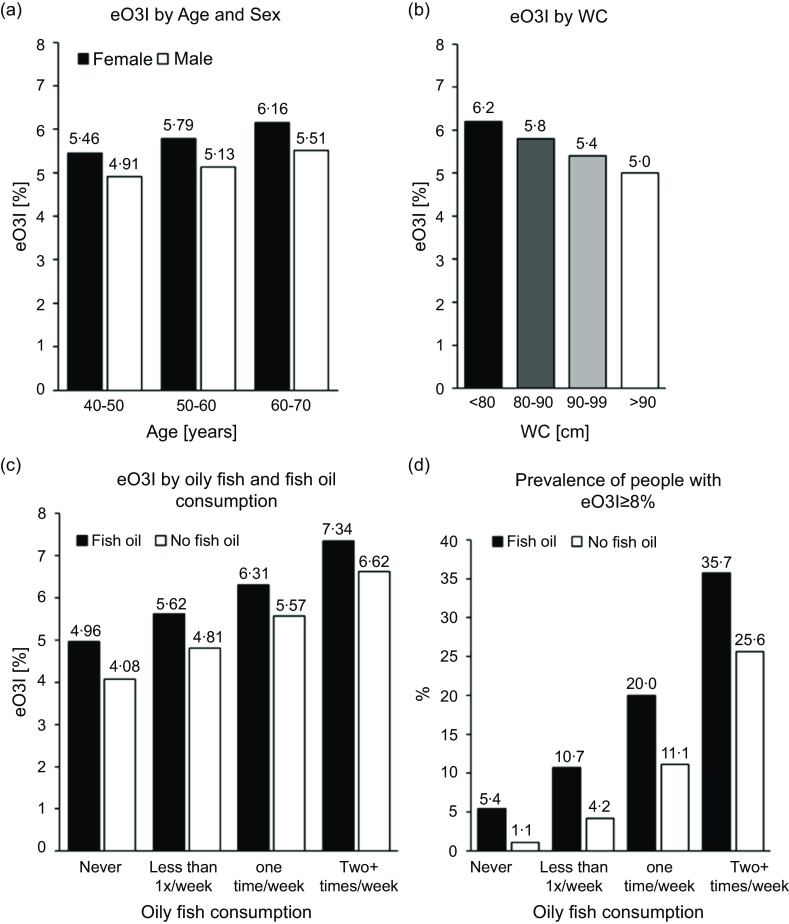
Predicted Omega-3 Index (eO3I) by (a) age and sex, (b) waist circumference (WC) and (c) oily fish and fish oil consumption. (d) Percentage of people with an eO3I ≥ 8 % depending on oily fish and fish oil consumption. All pairwise comparisons (male *v*. female in each age group, sex-specific age groups, all WC categories, fish oil *v*. no fish oil in different oily fish consumption groups and between different oily fish consumption groups) were *P* < 0·0001.

With the exceptions of rurality/urbanicity, virtually every metric included in Table 3 was statistically significantly correlated with the eO3I (*P* < 0·0001). For the *behaviour-independent* demographic variables, both sex and age were strongly associated with the eOI3 (Table 3 and Fig. 3(a)). The eO3I in men was 0·62 percentage points lower than in women before, and 0·38 % lower after adjustment. Additionally, the eO3I increased with age. Compared with the 40–50 year-age-group (reference group), the eO3I in the 50–60 year-, and 60–70 year-age-group increased by 0·29 and 0·64 %, respectively. After adjustment, the eO3I was still directly associated with age (0·14 and 0·34 %, respectively).

There was an inverse association between the eO3I and BMI, both before and after adjustment (Table 3). Compared with normal weight people, the mean eO3I was 0·4 and 0·88 % lower in the overweight and obese individuals, respectively. These differences were somewhat attenuated after adjustment but remained 0·13 or 0·38 %, respectively.

Associations between WC and eO3I were examined as a further body fat variable (Table 3 and Fig. 3(b)). Compared with the reference group, there was a monotonic decrease in the eO3I across WC quartiles. In the group with the highest WC, the eO3I was reduced by 1·13 % (adjusted, 0·55 %) compared with the reference group.

With regard to ethnic group, the eO3I in Blacks and Asians was not significantly different from that in Whites. ‘Other’ ethnic groups (3 % of the total) had higher eO3I than Whites (0·94 %, adjusted, 1·03 %).

SES (as reflected by the deprivation index) was inversely associated with the eO3I. Compared with the group of least deprived individuals, the eO3I decreased continuously as the deprivation index increased. The most deprived group showed a 0·13 % lower eO3I after adjustment compared with the least deprived group.

Another SES-related metric is the level of education. Compared to the group with the highest professional qualification (College, reference), the eO3I was lower in each successive group of educational achievement. At the extremes, individuals with the least education had a mean eO3I of 0·42 % (adjusted, 0·23 %) lower than the reference group.

As expected, regular consumption of oily fish and taking fish oil supplements were the strongest *behaviour-related* associations with the eO3I (Table 3, Fig. 3(c)). The mean eO3I of people who took fish oil and ate oily fish at least 2×/week (*n* 8279) was 7·34 %, but only 36 % of them had an eO3I ≥ 8 % (Fig. 3(d)). The fourteen characteristics examined in this study together explained about 21 % of the variability in the eO3I (Table 4). Regular oily fish intake alone explained 7 % of the 21 %, or about 34 % of the total. Compared with non-consumers of oily fish (reference), even less than one serving of oily fish per week was associated with a 0·77 % higher eO3I (0·47 % after adjustment). As oily fish consumption continued to increase, so did the eO3I, with 1 portion of oily fish per week being associated with a 1·56 % higher eO3I (adjusted, 1·11 %) and 2·64 % higher eO3I (adjusted, 2·12 %) with two or more portions of oily fish per week. Non-oily fish consumption was more weakly associated with the eO3I, explaining less than 1 % of the eO3I variability (Table 4). After adjustment, the difference in eO3I between those who report never eating non-oily fish and those consuming at least two portions of non-oily fish per week was only 0·49 %.

**Table 4.** *R* ^2^ values for the participant’s characteristics on the variability of eO3I (Percentages)

Overall model	20·5 %	Uniquely explained variability† %
Variable dropped*	New R* %	
Oily fish consumption	13·5	7·0
Regular fish oil consumption	18·7	1·8
Smoking	19·9	0·6
Ethnic group	19·9	0·6
Sex	20·1	0·4
Age	20·2	0·3
Alcohol use	20·2	0·3
Waist circumference	20·2	0·3
BMI	20·3	0·2
Education	20·3	0·2
Non-oily fish consumption	20·4	0·1
Exercise	20·4	0·1
Deprivation index	20·5	0·0
Urbanicity	20·5	0·0

* The new model *R* ^2^ for the remaining thirteen variables when predicting eO3I. Variables are ranked according to their independent contribution on the total variability.

† Uniquely explained variability is computed as the difference in the model without the variable compared with the full model with all fourteen variables (overall model).

On average, individuals who reported regular fish oil supplement use had a 0·97 % higher eO3I than individuals who did not. Even after adjustment for all the other factors in Table 3, it was still 0·68 % higher. The impact of fish oil supplementation on the eO3I was additive with the number of oily fish servings per week (Fig. 3(c)). People who reported taking a fish oil supplement and consuming two or more oily fish meals per week had a mean eO3I of 7·3 % (sd 2·7 %) compared with 4·1 % (sd 1·7 %) for those at the other extreme consuming neither (*P* < 0·0001). Fish oil intake explained ∼9 % of the total explained variability in the eO3I (Table 4).

Alcohol use also showed associations with the *n*3-PUFA biostatus. Compared to the group with the lowest alcohol use (none, reference group), the eO3I was increased with higher alcohol use. The highest alcohol use (daily) had a 0·4 % higher eO3I (adjusted, 0·3 %) compared with the reference group.

Smoking was negatively associated with the eO3I. Smokers had a 0·86 % lower eO3I compared with non-smokers, even after adjustment (0·58 %).

The level of exercise showed a weak but still significant inverse relationship to the eO3I. Compared to the group with the lowest level of physical activity (reference group), the eO3I was only lower in the group with the highest level of physical activity (adjusted, 0·15 %).

The plasma DHA% measured by NMR showed the same patterns/relationships with regard to all fourteen characteristics as did the eO3I (online Supplementary Table 1). However, all together, these predictors accounted for approximately 30 % of the variability in DHA% (online Supplementary Table S2), compared with only around 20 % for the eO3I (Table 4). For both DHA and eO3I, oily fish consumption and fish oil supplement use explained most of the variation.

## Discussion

In order to be able to investigate relationships between *n*3-PUFA and health or disease measures on populational level, it is preferable to use a biomarker of *n*3-PUFA status rather than an estimate of dietary intake from a questionnaire as recently recommended^(25)^. This is because biomarker levels are objectively measured, precise and reflect not only dietary intake but also *in vivo* metabolic conversions that cannot be captured with memory-based dietary intake surveys. The UK Biobank provides a large database to investigate such associations due to its extensive health data and number of participants. Since the O3I, which is a common metric to evaluate the *n*3-PUFA biostatus, was not measured directly in the UK Biobank, our goal was to develop a prediction model for the O3I in order to convert the data that do exist on *n*3-PUFA biostatus (plasma DHA% and total *n*3 % from NMR) into the eO3I. We found very good agreement (*r* = 0·82) between the estimated O3I values (eO3I) and the actual O3I. The eO3I equation was built on data from 242 samples, which were not included in the UK Biobank. We assumed, since the same lab (Nightingale) and method (NMR) were used for the UK Biobank analyses and for our inter-laboratory test, that the conversion equation should be applicable to UK Biobank data.

### Estimated Omega-3 Index in the UK Biobank in comparison with other countries

In the UK, there are no datasets from which a national, average O3I can be determined. A 2016 global survey reported ‘very low’ *n*3-PUFA levels in erythrocytes (< 4 % EPA + DHA of total FA) for the UK using data averaged from five published studies including a total of 461 individuals^(26)^. The mean eO3I in the UK Biobank cohort of 117 938 individuals was 5·58 %, and thus, it is significantly higher than that estimated by Stark *et al.* The eO3I in the UK Biobank is comparable to that of countries such as Germany (5·8 %) or the USA (5·44 %), as shown in a recent report^(27)^. Nevertheless, an average O3I of 5·58 % is still well below the optimal of ≥ 8 %. People who regularly ate oily fish and took supplemental fish oil had a significantly higher *n*3-PUFA biostatus. The mean eO3I of people who took fish oil and ate oily fish at least 2×/week (*n* 8279) was 7·34 % (sd 2·66 %), and 35·7 % (*n* 2957) of them had an eO3I ≥ 8 %. Thus, even among this group with the highest oily fish + supplement use, an optimal eO3I was not the norm.

### Predictors of the estimated Omega-3 Index in the UK Biobank cohort

Various factors besides EPA + DHA intake can affect O3I levels. Several other authors have explored this question^(28–31)^. Since not all comparative studies in the literature used the O3I as a metric, the general term ‘*n*3-PUFA biostatus’ is used below, which means the eO3I in relation to this study.

Consistent with our results, previous studies have already observed that female sex is positively associated with the *n*3 biostatus^(32–35)^. It has been suggested that a healthier diet (e.g. higher consumption of fish or plant *n*3-PUFA) and lifestyle (e.g. no smoking) could be a reason for the higher *n*3-PUFA biostatus in women^(33)^. However, the influence of factors such as smoking or (oily) fish and fish oil supplement intake and obesity were all considered in our model, and even after the adjustment there remained a significant and biologically relevant difference in the eO3I between the sexes. Other factors independent of lifestyle (e.g. genetics and metabolism) may also play a role. The reason for a higher *n*3-PUFA biostatus in females could be due to enhanced production of EPA + DHA from the precursor FA *α*-linolenic acid (C18:3), a conversion mediated at least in part by oestrogen^(36–38)^. In a series of studies, Burdge and Wootton^(37,39)^ concluded that oestrogen up-regulates delta-6 desaturase, which is the rate-limiting step in the conversion from *α*-linolenic acid to EPA and DHA. However, intakes of *n*3-PUFA were not controlled for over the entire study period, so a higher background consumption of preformed EPA + DHA cannot be ruled out^(40)^. In a study by Giltay *et al.* ^(36–38)^ in postmenopausal women who received hormone replacement therapy, the level of DHA in plasma cholesteryl esters increased by 20 %, which was attributed to the oestrogenic effect.

Although not addressed in this study, genotype could play a role in determining *n*3-PUFA biostatus. This, however, is controversial with some studies reporting an effect of a ‘heritability’ score^(33)^ on the O3I, whereas others found no association with the O3I of any given SNP^(41)^. We hope to investigate a possible genetic influence on the level of eO3I in future UK Biobank studies.

An age-related influence on the *n*3-PUFA biostatus is also known and has been established in several studies^(31,33,42)^. The reason for increased *n*3-PUFA biostatus in older individuals may be a function of decreased *n*3-PUFA turnover in tissues in older individuals^(43)^, but it does not appear to simply be the result of higher fish intake or supplement use as these were included in the adjusted model.

Our data are in line with comparable epidemiological studies which also found inverse associations between anthropometric markers such as the BMI^(44)^ or WC^(33,42)^ and *n*3-PUFA biostatus. In obese individuals, the eO3I in the present cohort was 0·38 % lower than that of normal weight individuals. The associations between WC and *n*3-PUFA biostatus appeared to be even stronger than those with BMI with a 0·55 % lower eO3I in the group with highest WC compared with the reference group. Since WC is a direct reflection specifically of abdominal obesity, WC is now thought to be superior to BMI as a predictor of cardio-metabolic diseases^(45)^. As to potential mechanisms, Cazolla *et al.* proposed that increased oxidative stress in obese individuals could lead to a reduction in the levels of EPA and DHA in erythrocyte membranes^(46)^. This was based on their observation that erythrocytes from obese individuals were more susceptible to oxidative stress than those from normal weight subjects. A disturbed hepatic PUFA metabolism may also contribute to a lower *n*3-PUFA biostatus. Animal studies found that obesity and diabetes resulted in reduced expression of key enzymes involved in the synthesis of EPA + DHA from *α*-linolenic acid^(47)^.

Several lifestyle variables were also associated with *n*3-PUFA biostatus. Consistent with the literature^(28,33,42,48–50)^, we found a strong negative influence of smoking on the *n*3-PUFA biostatus. Increased oxidative stress and lipid peroxidation, caused by smoking, may destroy long-chain PUFA such as EPA and DHA in cell membrane phospholipids and, thus, influence their levels in the body^(51,52)^. The possibility that this relationship is explained by differences in fish consumption and/or supplement use between smokers and non-smokers seems unlikely given the inclusion of these variables in the adjusted analysis. We also observed that the *n*3-PUFA biostatus was directly related to alcohol use, since the eO3I increased along with the reported frequency of alcohol use. Some studies also found alcohol use as an independent predictor of the *n*3-PUFA biostatus^(53)^, while others did not^(28,42)^. In the study by di Giuseppe, a positive influence of alcohol was only found in women and predominantly with wine drinking (and not beer or spirits). Since the UK Biobank does not provide information on the type of alcoholic beverages, a further differentiated analysis is not possible at this point. One can speculate that individuals who drink more alcohol may also eat more fish, and there was a modest association between oily fish eaters and drinking alcohol (e.g. 60 % of daily alcohol users eat oily fish at least once/week *v*. 50 % of monthly/hardly ever alcohol users). However, as with all of the potential determinants of the eO3I discussed here, significant associations with alcohol use remained after multivariable adjustment. In contrast, exercise in the UK Biobank cohort was only weakly associated with *n*3-PUFA biostatus. Small but significant differences were only found between the group with the highest and lowest reported minutes per week of physical exercise. In line with our results, a previous study^(54)^ found significantly lower O3I levels in German national elite winter endurance athletes (mean O3I: 4·97 %) or National Collegiate Athletic Association Division I athletes in the USA (mean O3I: 4·33 %)^(55)^ compared with the general population in Germany (mean O3I: 5·8 %) and the USA (mean O3I: 5·44 %)^(27)^. In contrast to our results, a cross-sectional study showed that exercise time, exercise capacity and heart rate recovery directly correlated with the O3I in patients with coronary artery disease^(56)^. At present, it is not clear why intense exercise may be associated with lower *n*3-PUFA status.

The consumption of preformed EPA and DHA has the greatest influence on the *n*3-PUFA biostatus, as various other studies have already shown^(28,33,42,44)^. The highest *v*. the lowest oily fish intake was associated with an unadjusted increase in the eO3I of 2·6 %, decreasing only to 2·2 % after adjustment. Fish oil supplement use had the second greatest effect in our study (i.e. 1 % increase; 0·67 % adjusted). The adjusted value was approximately equal to that (0·5 %) of consuming < 4 servings of oily fish per month. Since daily consumption of the most generic fish oil supplement would provide about 300 mg of EPA + DHA per day (about 2100 mg per week), and one serving of an oily fish like salmon would provide roughly 1000 mg of EPA + DHA per week, the smaller effect of the eO3I of reported supplement use here suggests that the respondents in this cohort may have either overestimated what ‘regular’ fish oil consumption means or underestimated their fish intake. These limitations are elaborated on below. Suffice it to say, that as noted above, people reporting both > 2 oily fish meals per week AND fish oil supplement use had an adjusted eO3I of 7·34 % compared with the 4·08 % of the reference group consuming no fish or supplements.

The deprivation index – a measure of SES – was also identified as an inverse and independent predictor of the *n*3-PUFA biostatus confirming past studies^(33)^. Individuals with a higher SES tend to behave healthier, have a lower BMI and smoke less^(57)^, and they have a healthier diet, which also includes higher fish and seafood consumption^(58,59)^. However, several of these factors were considered in our adjusted model, and the inverse relationship between the eO3I and the deprivation index remained significant. Statistical modelling can never fully account for the myriad of behavioural, environmental and dietary factors associated with differences in SES. We also identified education as a strong independent predictor for eO3I as have others^(42)^. The association between education and the eO3I was still significant even after adjustment for the deprivation index.

### Strength and limitations

The strength of the study is clearly the very large and fairly well-characterised cohort and an objectively measured biomarker of *n*3-PUFA status. However, the study has a number of potential limitations. First, the eO3I had to be extrapolated from plasma *n*3-PUFA measures done by NMR. The imprecision arising from this extrapolation (accounted for with multiple imputations of the eO3I) diminished the ability of the suite of variables we included to predict *n*3-PUFA biostatus. Specifically, the fourteen factors explained 30 % of the variability in plasma DHA% but only 20 % of the eO3I variability. Thus, some relationships may have been missed as a result of using the eO3I. Another limitation is the relatively narrow age range of participants, from 40 to 70 years. Thus, our data on the eO3I cannot be extrapolated to other age groups. Obviously, these data only apply to the UK or perhaps ‘western’ populations in general (given the similarity in eO3I values seen here and in other countries^(27)^), but they do not apply to countries with diets and lifestyles differing markedly from those in the UK.

Finally, self-reported fish consumption data are prone to under- and over-reporting. Given a general understanding of ‘fish as a healthy food’, people are more likely to over-report their fish consumption. In the UK Biobank study, no portion sizes of oily and non-oily fish consumption were collected which could, theoretically, have allowed more precise associations between fish intake and eO3I to be observed. In addition, no other data on fish oil supplement intake were collected (e.g. dosage, potency and frequency of intake). These variables therefore only provided a rough estimate of the actual consumption of fish and fish oil. As usual, data from cross-sectional studies cannot reveal causal relationships. Our data are thus only hypothesis generating.

### Conclusion

The results of this investigation allow for the first time an estimation of the average O3I in the UK. Second, they largely confirm previously observed predictors of the O3I, namely oily fish consumption, fish oil intake, sex, age, WC, BMI and various lifestyle variables. A recent study with the UK Biobank showed that fish oil supplementation was associated with reduced risk for CVD outcomes and all-cause mortality^(60)^. With the ability to derive an eO3I as a metric for the *n*3-PUFA biostatus, it will now be possible to investigate its relationship with risk for multiple diseases such as CVD, type 2 diabetes mellitus or neurodegenerative disorders in the UK Biobank.

## Supplementary material

For supplementary material accompanying this paper visit https://doi.org/10.1017/S0007114522003282. click here to view supplementary material
