# Supplementary material for: Estimation and predictors of the Omega-3 Index in the UK Biobank
Source: Br J Nutr. 2022 Oct 10;130(2):312–22. doi: 10.1017/S0007114522003282 (PMC10277661; doi:10.1017/S0007114522003282)
Supplement: Supplementary file 1 [file S0007114522003282sup001.docx]

**Determinants of the Omega-3 Index in the UK Biobank**

**Supplemental Material**

**Supplemental Table 1: Associations of demographic characteristics and DHA% (measured by NMR) in unadjusted and adjusted (for all variables in Table 1) analyses. (n=117,108).**

| **Characteristics** | **DHA%**  **Mean (SD)** | **DHA%**  **Unadj Diff or Corr (95% CI)** | **Unadj P-value** | **DHA%**  **Adj Diff or Corr (95% CI)** | **Adj P-value** |
| --- | --- | --- | --- | --- | --- |
| **Sex** |  |  |  |  |  |
| Female | 2.1 (0.7) | 0 |  | 0 |  |
| Male | 1.9 (0.7) | -0.24 (-0.25,-0.24) | <0.0001*** | -0.13 (-0.14,-0.12) | <0.0001*** |
| **Age** (years) | |  |  |  |  |
| 40-50 (reference) | 1.9 (0.6) | 0 |  | 0 |  |
| 50-60 | 2 (0.7) | 0.07 (0.06,0.08) | <0.0001*** | 0.03 (0.02,0.03) | <0.0001*** |
| 60-70 | 2.1 (0.7) | 0.17 (0.16,0.18) | <0.0001*** | 0.09 (0.08,0.09) | <0.0001*** |
| **BMI** (kg/m^2^) | |  |  |  |  |
| 18.5-24.9 (normal weight; reference) | 2.2 (0.7) | 0 |  | 0 |  |
| 25.0-29.9 (overweight) | 2 (0.7) | -0.19 (-0.2,-0.19) | <0.0001*** | -0.07 (-0.08,-0.06) | <0.0001*** |
| ≥30 (obese) | 1.8 (0.6) | -0.4 (-0.41,-0.39) | <0.0001*** | -0.17 (-0.18,-0.16) | <0.0001*** |
| **Waist Circumference** (cm) |  |  |  |  |  |
| <80 (reference) | 2.3 (0.7) | 0 |  | 0 |  |
| 80-90 | 2.1 (0.7) | -0.18 (-0.19,-0.17) | <0.0001*** | -0.11 (-0.12,-0.1) | <0.0001*** |
| 90-99 | 1.9 (0.7) | -0.33 (-0.34,-0.32) | <0.0001*** | -0.18 (-0.19,-0.17) | <0.0001*** |
| >99 | 1.8 (0.6) | -0.5 (-0.51,-0.49) | <0.0001*** | -0.26 (-0.28,-0.24) | <0.0001*** |
| Do not know/No answer | 1.7 (0.5) | -0.55 (-0.79,-0.32) | <0.0001*** | -0.37 (-0.58,-0.17) | 0.00035** |
| **Ethnic group** |  |  |  |  |  |
| White (reference) | 2 (0.7) | 0 |  | 0 |  |
| Black | 2.1 (0.7) | 0.06 (0,0.11) | 0.034* | 0.07 (0.02,0.11) | 0.003** |
| Asian | 1.8 (0.7) | -0.21 (-0.24,-0.18) | <0.0001*** | -0.01 (-0.04,0.01) | 0.43 |
| Other | 2.4 (0.8) | 0.35 (0.33,0.37) | <0.0001*** | 0.38 (0.36,0.4) | <0.0001*** |
| **Deprivation index** |  |  |  |  |  |
| Least Deprived (reference) | 2.1 (0.7) | 0 |  | 0 |  |
| Next least deprived | 2 (0.7) | -0.03 (-0.04,-0.02) | <0.0001*** | 0 (-0.01,0.01) | 0.56 |
| Typical Deprivation | 2 (0.7) | -0.07 (-0.08,-0.05) | <0.0001*** | -0.02 (-0.03,-0.01) | <0.0001*** |
| Somewhat deprived | 1.9 (0.7) | -0.11 (-0.13,-0.1) | <0.0001*** | -0.04 (-0.05,-0.03) | <0.0001*** |
| Most deprived | 1.9 (0.7) | -0.14 (-0.16,-0.13) | <0.0001*** | -0.04 (-0.05,-0.02) | <0.0001*** |
| **Urbanicity** |  |  |  |  |  |
| England/Wales- Urban (reference) | 2 (0.7) | 0 |  | 0 |  |
| England/Wales – Rural | 2.1 (0.7) | 0.08 (0.06,0.1) | <0.0001*** | 0.01 (-0.01,0.02) | 0.23 |
| Scotland – Rural | 2 (0.7) | 0 (-0.04,0.05) | 0.86 | 0.01 (-0.03,0.05) | 0.59 |
| Scotland – Urban | 2 (0.7) | -0.02 (-0.04,-0.01) | 0.0024** | -0.02 (-0.03,0) | 0.028* |
| **Education** |  |  |  |  |  |
| College (reference) | 2.1 (0.7) | 0 |  | 0 |  |
| Associates | 2 (0.7) | -0.05 (-0.07,-0.04) | <0.0001*** | -0.03 (-0.04,-0.02) | <0.0001*** |
| SEs | 2 (0.7) | -0.14 (-0.15,-0.13) | <0.0001*** | -0.07 (-0.08,-0.06) | <0.0001*** |
| None | 1.9 (0.7) | -0.18 (-0.19,-0.17) | <0.0001*** | -0.09 (-0.1,-0.08) | <0.0001*** |
| Do not know/No answer | 1.9 (0.6) | -0.17 (-0.21,-0.13) | <0.0001*** | -0.11 (-0.14,-0.07) | <0.0001*** |
| **Oily fish consumption** |  |  |  |  |  |
| Never (reference) | 1.6 (0.5) | 0 |  | 0 |  |
| Less than 1x/week | 1.8 (0.5) | 0.26 (0.25,0.27) | <0.0001*** | 0.16 (0.15,0.17) | <0.0001*** |
| 1x/week | 2.1 (0.6) | 0.53 (0.52,0.55) | <0.0001*** | 0.38 (0.37,0.4) | <0.0001*** |
| At least 2x/week | 2.5 (0.8) | 0.9 (0.89,0.92) | <0.0001*** | 0.73 (0.71,0.74) | <0.0001*** |
| **Non-oily fish consumption** |  |  |  |  |  |
| Never (reference) | 1.6 (0.6) | 0 |  | 0 |  |
| Less than 1x/week | 1.9 (0.6) | 0.33 (0.31,0.35) | <0.0001*** | 0.11 (0.1,0.13) | <0.0001*** |
| 1x/week | 2 (0.7) | 0.46 (0.44,0.48) | <0.0001*** | 0.11 (0.09,0.13) | <0.0001*** |
| At least 2x/week | 2.2 (0.7) | 0.6 (0.58,0.62) | <0.0001*** | 0.16 (0.14,0.18) | <0.0001*** |
| **Regular Fish oil consumption** |  |  |  |  |  |
| No (reference) | 1.9 (0.6) | 0 |  | 0 |  |
| Yes | 2.2 (0.7) | 0.32 (0.31,0.33) | <0.0001*** | 0.22 (0.21,0.23) | <0.0001*** |
| **Alcohol use** |  |  |  |  |  |
| Rarely/Never (reference) | 1.9 (0.7) | 0 |  | 0 |  |
| 1-3x/month | 1.9 (0.7) | 0 (-0.01,0.01) | 0.95 | 0 (-0.01,0.02) | 0.5 |
| 1-2x/week | 2 (0.7) | 0.06 (0.05,0.07) | <0.0001*** | 0.04 (0.03,0.05) | <0.0001*** |
| 3-4x/week | 2.1 (0.7) | 0.15 (0.13,0.16) | <0.0001*** | - 1. (0.09,0.11) | <0.0001*** |
| Daily | 2.1 (0.7) | 0.15 (0.14,0.16) | <0.0001*** | 0.12 (0.1,0.13) | <0.0001*** |
| **Smoker** |  |  |  |  |  |
| Never (reference) | 2.1 (0.7) | 0 |  | 0 |  |
| Previous | 2 (0.7) | -0.03 (-0.04,-0.02) | <0.0001*** | -0.01 (-0.02,-0.01) | 0.00074** |
| Current | 1.7 (0.6) | -0.31 (-0.32,-0.3) | <0.0001*** | -0.21 (-0.22,-0.2) | <0.0001*** |
| **Exercise** (min/week) |  |  |  |  |  |
| <150 (reference) | 2 (0.7) | 0 |  | 0 |  |
| 150-320 | 2 (0.7) | 0.09 (0.09,0.1) | <0.0001*** | 0 (0.,0.02) | 0.2 |
| 320-660 | 2.1 (0.7) | 0.1 (0.09,0.12) | <0.0001*** | -0.01 (-0.02,0) | 0.23 |
| >660 | 2 (0.7) | 0.06 (0.05,0.07) | <0.0001*** | -0.04 (-0.05,-0.03) | <0.0001*** |
| Do not know/No answer | 1.9 (0.7) | -0.04 (-0.06,-0.03) | <0.0001*** | -0.03 (-0.05,-0.02) | <0.0001*** |

**Supplemental Table 2: R-squared values for the participant’s characteristics on the variability of DHA% measure by NMR.**

| **Overall model** | 29.2% |  |  |  |
| --- | --- | --- | --- | --- |
| **Variable dropped^1^** | **New R-squared^1^** | | **Uniquely explained variability^2^** | |
| **Oily fish consumption** | 20.1% |  | 9.1% |  |
| **Regular fish oil consumption** | 27.0% |  | 2.2% |  |
| **Ethnic group** | 28.4% |  | 0.9% |  |
| **Smoking** | 28.4% |  | 0.8% |  |
| **Waist Circumference** | 28.6% |  | 0.6% |  |
| **Sex** | 28.6% |  | 0.6% |  |
| **Alcohol use** | 28.8% |  | 0.4% |  |
| **BMI** | 28.8% |  | 0.4% |  |
| **Education** | 29.0% |  | 0.3% |  |
| **Age** | 29.0% |  | 0.2% |  |
| **Non-oily fish consumption** | 29.0% |  | 0.2% |  |
| **Exercise** | 29.1% |  | 0.1% |  |
| **Urbanicity** | 29.1% |  | 0.1% |  |
| **Deprivation index** | 29.2% |  | 0.1% |  |

1. New R-squared provides the model R-squared for the other 13 variables when predicting DHA%. Variables are ranked according to their independent contribution on the total variability.

2. Uniquely explained variability is computed as the difference in the model without the variable compared to the full model with all 14 variables (overall model).

| **SF 1a. Histogram of residuals**  **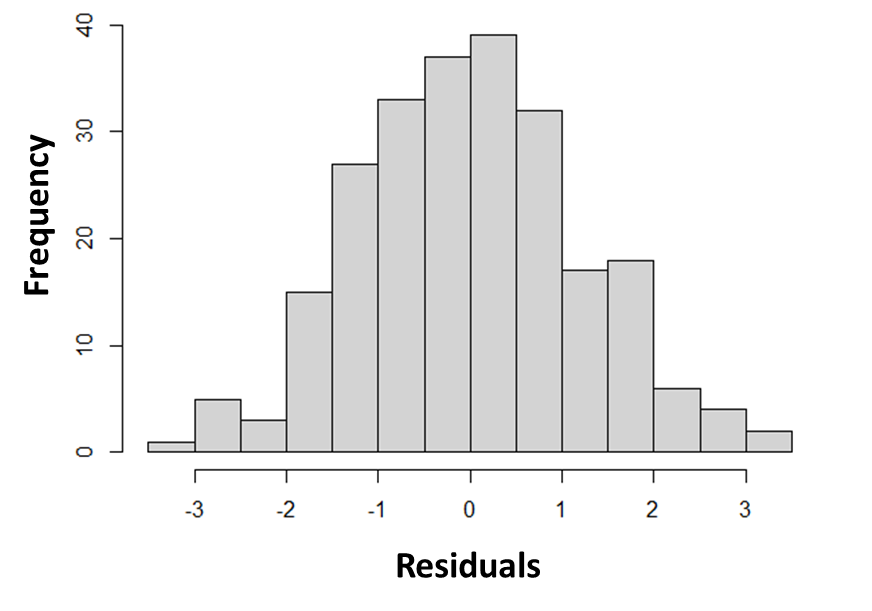** | **SF 1b. Residuals vs DHA% (NMR)**  **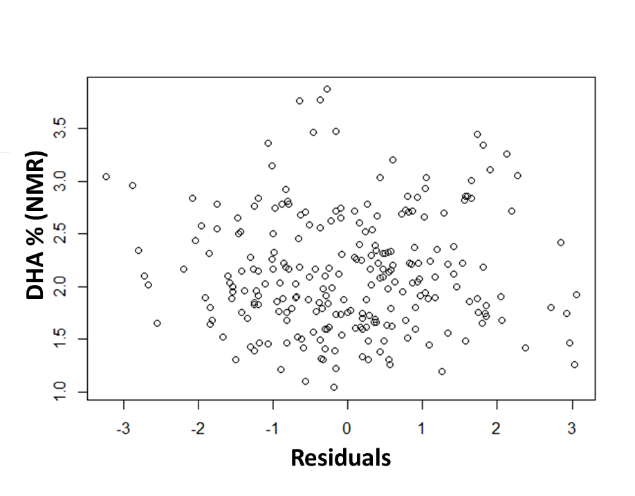** |
| --- | --- |
| **SF 1c. Residuals vs non-DHA% (NMR)**  **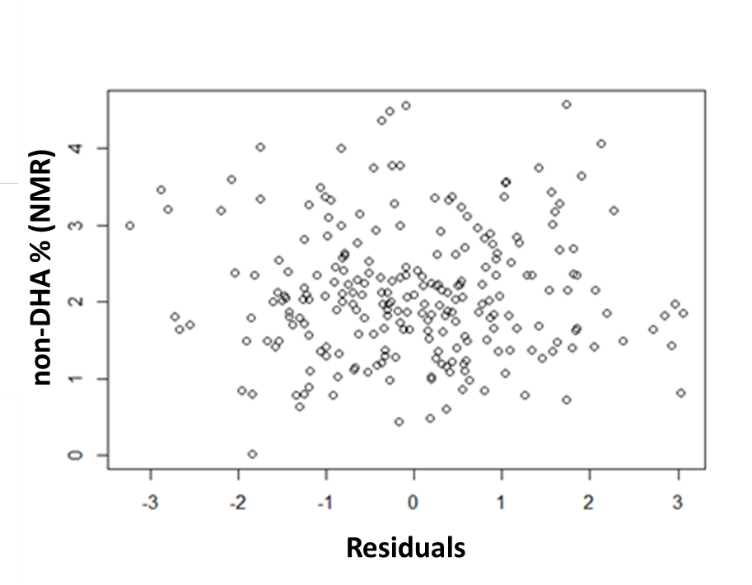** |  |

**Supplemental Figure 1: Prediction model diagnostics.**

Figure 1a shows the normality of the residuals for the final prediction model. Figures 1b and 1c show good model fit characteristics when looking at the prediction model residuals vs both predictor variables (DHA% [Figure 1b]; non-DHA% [Figure 1c]).
